# First person – Pranidhi Baddam

**DOI:** 10.1242/dmm.048914

**Published:** 2021-02-11

## Abstract

First Person is a series of interviews with the first authors of a selection of papers published in Disease Models & Mechanisms, helping early-career researchers promote themselves alongside their papers. Pranidhi Baddam is first author on ‘Neural crest-specific loss of *Bmp7* leads to midfacial hypoplasia, nasal airway obstruction and disordered breathing, modeling obstructive sleep apnea’, published in DMM. Pranidhi is a PhD student in the lab of Daniel Graf at the University of Alberta, Edmonton, Canada, investigating the contribution of different types of cartilage to midfacial growth.


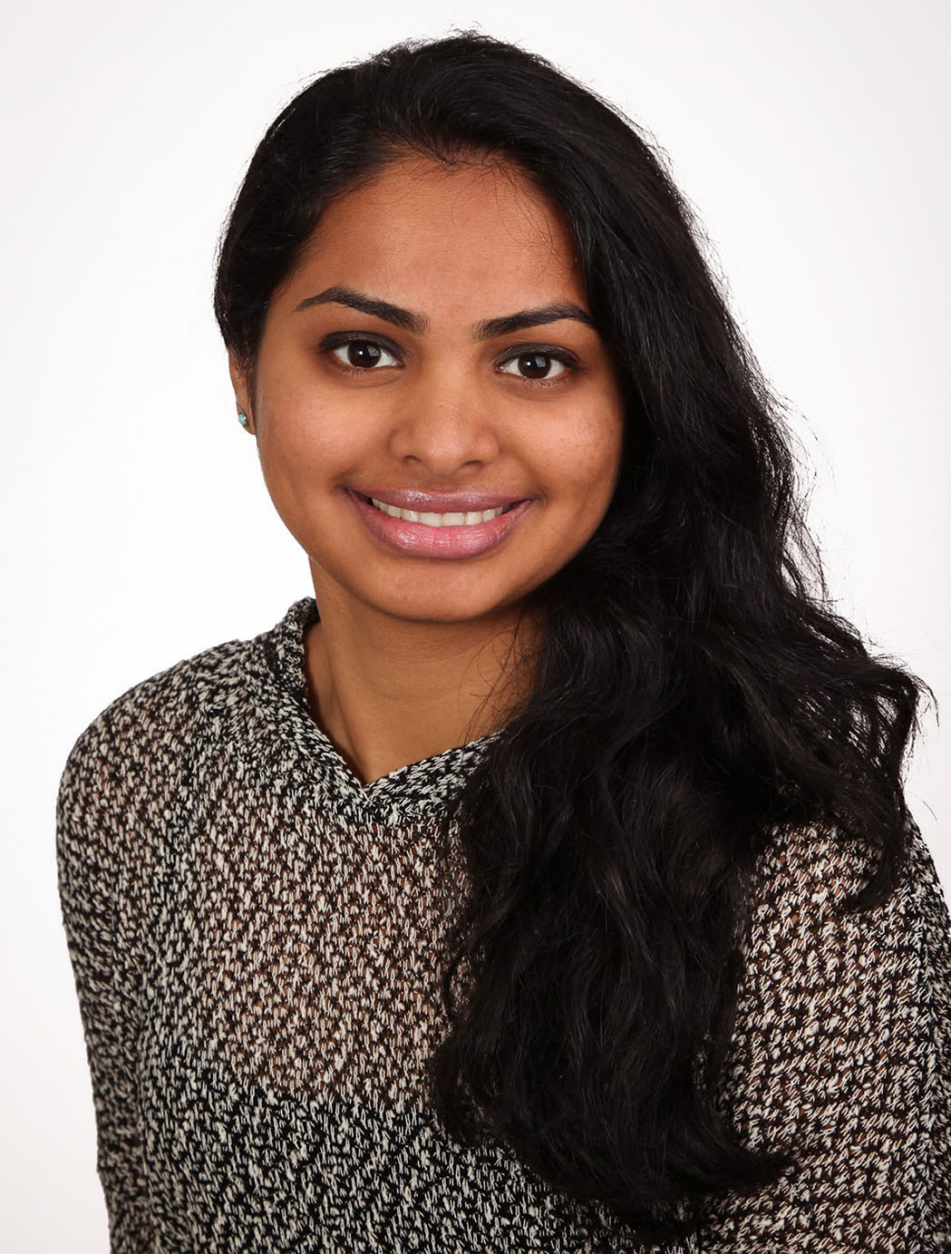


**Pranidhi Baddam**

**How would you explain the main findings of your paper to non-scientific family and friends?**

Paediatric obstructive sleep apnoea (OSA) is a common sleep-related breathing disorder (SRBD) observed in children. This condition results from physical blockage of the upper nasal or pharyngeal airways. Currently, it is not possible to predict whether and when a child with midfacial hypoplasia will develop a SRBD. Up to now, a rodent mouse model that investigates how facial abnormalities result in nasal airway obstruction and its associated secondary complications did not exist. In our paper, we show that mice with a deletion of a gene called *Bmp7* in cells that form the face results in lack of midfacial growth, nasal septum deviation and disordered breathing. We found that many of these abnormalities develop after birth and during a period of rapid midfacial growth, before breathing disturbances develop. Overall, our data show that the mutant mice recapitulate many clinical features observed in children with OSA, including lack of midfacial growth, nasal septum deviation and breathing disturbances.

“The appearance of nasal septum deviation at a late stage of craniofacial growth is of great interest.”

**What are the potential implications of these results for your field of research?**

The appearance of nasal septum deviation at a late stage of craniofacial growth is of great interest. What is the cause? Could it be that developmental changes predispose the nasal cartilage to deviate? I am currently pursuing this particular aspect. Given that the nasal cartilage is a hyaline cartilage, a type of cartilage that is resilient to compression, a deviation should not occur. Using this model, I hope to find a molecular explanation for the deviation.

**What are the main advantages and drawbacks of the model system you have used as it relates to the disease you are investigating?**

The main advantage of this mouse model is that it recapitulates many features observed in children with OSA and offers a unique opportunity to study how upper nasal airway obstruction affects breathing physiology and leads to systemic morbidities. A drawback of this model is that it doesn't easily allow for long-term assessment of the breathing once nasal septum deviation is established as most of these mice die due to secondary comorbidities. Additionally, we have identified that no single craniofacial abnormality in this model appeared to predict upper-airway obstruction.

**What has surprised you the most while conducting your research?**

This transgenic mouse model is a genetically defined mouse model that is housed in a stable environment. So I was surprised to see variability in breathing within the mutant mice, although all mice develop nasal septum deviation. On reflection, this makes this mouse model unique as it recapitulates the complexity of features observed in children with SRBDs.

**Figure:**
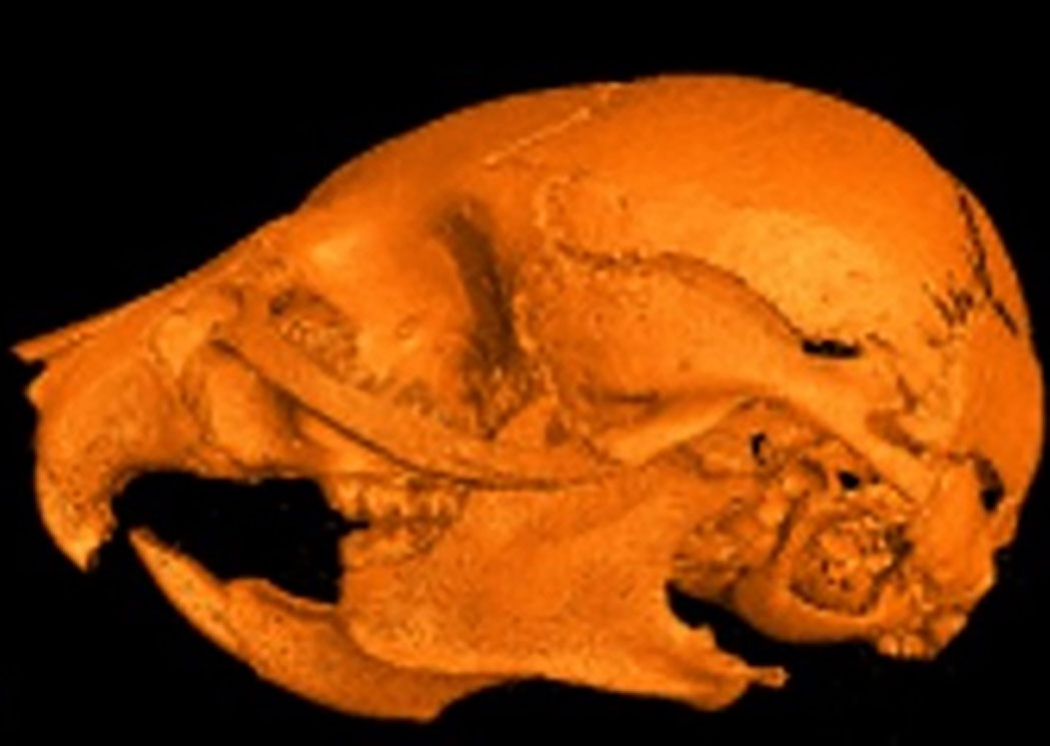
**Micro-computed tomography reconstruction of a 1-month-old Bmp7^ncko^ mouse demonstrating facial abnormalities.**

**Describe what you think is the most significant challenge impacting your research at this time and how will this be addressed over the next 10 years?**

The most significant challenge that clinicians and researchers investigating nasal airway obstruction and SRBD face is the ability to determine/diagnose using computed tomography scans whether children with craniofacial abnormalities will develop nasal obstruction and SRBD and require further intervention. Establishing better diagnostic criteria such that children with nasal obstruction can be stratified into subcategories based on symptom presentation may result in efficient triaging process. Additionally, using genetic animal models like the one in this study to understand how craniofacial structures grow, repair and interact with surrounding tissues will contribute to the development of precision-based diagnosis and treatment. Moreover, investigating environmental factors that contribute to craniofacial abnormalities like midfacial hypoplasia and nasal septum deviation will also assist in predicting the onset and severity of nasal airway obstruction.

**What changes do you think could improve the professional lives of early-career scientists?**

As early-career professionals take on the journey of becoming independent scientists, some changes that can be implemented would be better funding support, fostering a collaborative environment, access to cutting-edge technology and mentorship. Additionally, early-career scientists could also have flexibility without being overly stressed about acquiring funding when investigating topics that don't directly impact human health but advance knowledge in foundational sciences.

**What's next for you?**

I am currently finishing my PhD and in pursuit of a postdoc position. I would like to contribute to aspects of cartilage tissue engineering by expanding my understanding of how craniofacial cartilages are derived.
